# Ministernotomy reduces intubation time in aortic valve replacement with Perceval prosthesis

**DOI:** 10.1186/1749-8090-10-S1-A61

**Published:** 2015-12-16

**Authors:** Emmanuel Villa, Antonio Messina, Margherita Dalla Tomba, Federico Brunelli, Marco Cirillo, Zen Mhagna, Alessandra D'Agostino, Giovanni Troise

**Affiliations:** 1 Cardiac Surgery, Poliambulanza Foundation Hospital, Brescia, 25124, Italy; 2 Catholic University, Rome, 00168, Italy

## Background/Introduction

Sutureless technology for aortic valve replacement (AVR) seems to reduce morbidity/mortality and minimally-invasive procedures are supposed to be facilitated. Consequently, even better results are expected from minimally-invasive surgery, but evidence of improvement is scarce.

## Aims/Objectives

To shed more light in this field, we studied the effect on hospital outcome of ministernotomy (MS) versus full sternotomy (FS) in AVR with Sorin Perceval.

## Method

From a single-center prospective registry (period 3/2011-2/2015), 104 patients underwent Perceval AVR without associated procedures. Three presented with absolute contraindications to MS and was discarded to favor a propensity score analysis. Accordingly, 67 with FS and 34 with a reversed-T MS were available. A logistic regression was performed and a nearest neighbor matching gave 24 couples.

## Results

Preoperative profile was similar in FS and MS: mean age 81+/-4.1 vs 81.2+/-4 (p = 0.91), BMI 27.4+/-5.5 vs 27.2+/-4.2 (p = 0.97), COPD 4.2% vs 4.2%, creatinine 0.97+/-0.31 vs 1+/-0.23 mg/dl (p = 0.38), diabetes 33.3% vs 41.7% (p = 0.77), EF 0.61 +/-0.11 vs 0.62+/-0.7 (p = 0.73), median frailty index 1 (IQR 0-3) vs 1 (IQR 0.5-2) (p = 0.65). FS assured faster operative times than MS: CPB 70.9+/-15.8 vs 86+/-16.5 (p = 0.002), cross-clamp 46.2+/-12.3 vs 58+/-12.6 min (p = 0.002). However, median intubation time was longer in FS (8 hours, 7-11) respect to MS (7 hours, 5-10.5) (p = 0.021). Hospital outcome did not differ: mortality 8.3% vs 0 (p = 0.49), re-exploration 4.2% vs 0 (p = 1), sternal dehiscence 4.2% vs 0 (p = 1), a-fib 45.8% vs 58.3% (p = 0.76), pacemaker 4.2% vs 8.3% (p = 1), median postop stay 6 vs 7 days (p = 0.23).

## Discussion/Conclusion

Although MS required longer CPB/cross-clamp times, intubation was shorter. Other clinical benefits from MS-AVR demands larger cohorts to be demonstrated.

